# Complete Genome Sequence of *Bacillus* sp. Strain V3, Isolated from Mangrove Sediments in Wenzhou, China

**DOI:** 10.1128/MRA.01448-20

**Published:** 2021-05-06

**Authors:** Ruihua Yan, Shija Vicent Michael, Guangya Zhang, Runying Zeng, Xialan Li

**Affiliations:** a Department of Biotechnology and Bioengineering, Huaqiao University, Xiamen, China; b Technology Innovation Center for Exploitation of Marine Biological Resources, Third Institute of Oceanography, Ministry of Natural Resources, Xiamen, China; c Department of Marine Science and Technology, Zhejiang Ocean University, Zhoushan, China; University of Maryland School of Medicine

## Abstract

We present the complete genome sequence of *Bacillus* sp. strain V3, which was isolated from mangrove sediments from Yanpu Bay, Wenzhou, China. The 4.44-Mb genome sequence of V3 will be useful for studying the functional genes with potential industrial application of this species from mangrove sediments.

## ANNOUNCEMENT

In this study, *Bacillus* sp. strain V3, with pullulanase activity, was isolated from mangrove sediments collected from Yanpu Bay in Wenzhou City, China. This work aimed to sequence the complete genome of strain V3, which will be useful for exploring the pullulanase gene in *Bacillus* sp. strain V3 and explaining the ecological role of strain V3 in mangroves. Isolation was performed using the standard dilution plating method with marine agar 2216E (1). After 24 h of aerobic incubation at 28°C, an isolated colony was picked and purified by repeated streaking onto marine agar 2216E. The genomic DNA of strain V3 in the same culture broth was extracted three times at room temperature with a bacterial DNA extraction kit (Tiangen, China) to ensure the amount and purity of DNA required for construction of the sequencing library. The DNA extracts were pooled, and the purity, concentration, and integrity were examined with a NanoDrop spectrophotometer (Thermo Fisher Scientific, Massachusetts), Qubit fluorometer (Thermo Fisher Scientific), and 1% agarose gel electrophoresis, respectively. The genomic sequencing was performed by Biomarker Technologies Ltd. (China) using the Nanopore sequencing platform according to the standard procedure (2) from Oxford Nanopore Technologies (UK).

The software that was used to analyze the genome of strain V3 was run with default parameters unless otherwise specified. The 16S rRNA gene sequence in the genome of V3 was extracted by ContEst16S (3). After a sequence BLAST (v2.0) search in EzBioCloud (4), strain V3 was assigned as a *Bacillus* species based on its 16S rRNA gene, with a maximum identity of 99.39%. The depth of genomic sequencing was 384.12×, and the complete genome size of strain V3 was 4.44 Mbp. The drop-off data format for Nanopore sequencing was the binary Fast5 format. The Fast5 data were converted to the fastq format after base calling by Albacore software in the MinKNOW (v20.06.18) software package. A clean data set was obtained after further filtering of duplicate, low-quality (quality score,  <6), and short (length, <2,000 bp) reads by using a perl script named "kmer freq stat" that was devoloped by Biomarker Technologies Ltd. A total of 131,325 clean reads with 1,709,310,082 bp and an *N*_50_ value of 19,467 bp were obtained. The genome was assembled (with Canu v1.5 [correctedErrorRate = 0.120, corCoverage = 40]) (5) and polished (with Pilon v1.22) (6) into one contig with a length of 4,449,890 bp and a GC content of 44.53% by using Nanopore reads. The assembled genome was then cyclized using the Minimus2 cyclization process in Circlator (v1.5.5) (7). Genome annotation was performed with the NCBI Prokaryotic Genome Annotation Pipeline (PGAP) (v5.0) (8–10). Totals of 4,306 coding genes, 27 rRNAs (9, 9, and 9 genes for 5S rRNA, 16S rRNA, and 23S rRNA, respectively), 90 tRNAs, and 5 noncoding RNAs were annotated in the genome of V3. The genes for α-amylase and pullulanase were annotated in the genome of *Bacillus* sp. strain V3, which indicates a role for strain V3 in the degradation of starch macromolecular polysaccharide and the potential industrial application of this strain.

### Data availability.

The complete genome sequence of *Bacillus* sp. strain V3 has been deposited in GenBank under the accession number CP065544. The SRA records are available under the accession numbers SRX9818096 and SRX9818097. The BioSample and BioProject accession numbers are SAMN17005973 and PRJNA682715, respectively.
